# Mortality among persons with tuberculosis in Zambian hospitals: A retrospective cohort study

**DOI:** 10.1371/journal.pgph.0003329

**Published:** 2024-06-17

**Authors:** Josphat Bwembya, Ramya Kumar, Victoria Musonda, Rhehab Chimzizi, Nancy Kasese-Chanda, Lameck Goma, Mushota Kabaso, Reford Mihova, Sulani Nyimbili, Vimbai Makwambeni, Soka Nyirenda, Alwyn Mwinga, Patrick Lungu

**Affiliations:** 1 Eradicate TB, PATH, Lusaka, Zambia; 2 Zambart, UNZA Ridgeway, Lusaka, Zambia; 3 Ministry of Health, Lusaka, Zambia; 4 USAID STAR Project, Lusaka, Zambia; 5 USAID, Lusaka, Zambia; Pontificia Universidad Católica de Chile: Pontificia Universidad Catolica de Chile, CHILE

## Abstract

Tuberculosis (TB) mortality in Zambia remains high at 86 per 100,000 populations, translating to approximately 15,000 TB-related deaths annually. We conducted a nationwide retrospective cohort study to understand predictors, time to death, and probable causes of mortality among persons on TB treatment in Zambia. We reviewed medical records for persons with TB registered in 54 purposively selected hospitals in Zambia between January and December 2019. We fitted a Cox proportional hazards model to identify predictors of mortality. Of the 13,220 records abstracted, 10,987 were analyzed after excluding records of persons who transferred in from other hospitals, those with inconsistent dates and those whose treatment outcome was not evaluated. The majority of persons with TB were men, (61.5%, n = 6,761) with a median age of 36 years (IQR: 27–46 years). Overall, 1,063 (9.7%) died before completing TB treatment (incidence rate = 16.9 deaths per 1,000 person-months). Median age at death was 40 years (IQR: 31–52). The majority of deaths (75.7%, n = 799) occurred in the first two months of TB treatment, with a median time to death of 21 days (IQR: 6–57). Independent risk factors for TB mortality included age >54 years, being treated in Eastern, Southern, Western, Muchinga and Central provinces, receiving treatment from a third-level or mission hospital, methods of diagnosis other than Xpert MTB/RIF, extrapulmonary TB (EPTB), and positive HIV status. Probable causes of death were septic shock (18.8%), TB Immune Reconstitution Inflammatory Syndrome (TB IRIS) (17.8%), end-organ damage (13.4%), pulmonary TB (11.4%), anemia (9.6%) and TB meningitis (7.8%). These results show high mortality among people undergoing TB treatment in Zambia. Interventions targeted at persons most at risk such as the elderly, those with EPTB, and those living with HIV, can help reduce TB-related mortalities in Zambia.

## Introduction

In 2022, the World Health Organization (WHO) ranked tuberculosis (TB) among the top 10 causes of death worldwide and a leading cause of death from a single infectious agent (ranking above HIV/AIDS) [1]. Globally, an estimated 10.6 million people developed TB disease in 2021. In the same period, an estimated 1.4 million TB-related deaths were recorded among HIV-negative people, and an additional 187,000 deaths were recorded among HIV-positive people [1]. Nine out of ten tuberculosis deaths occur in high-burden countries, particularly Sub-Saharan Africa [2].

Although Zambia is among the high-burden countries in Southern Africa that have recorded an impressive reduction in TB mortality rate from 228 per 100,000 populations in 2000 to 86 per 100,000 populations in 2020, this rate remains high [1, 3]. Further, the TB program conducted an epidemiological review of their routine programme data in 2020 which revealed that since 2013, the mortality rate for all forms of TB has been oscillating around 6%, ranging from 3.3% for Lusaka Province, the country’s largest and capital city, to as high as 13.6% for Southern Province [4].

Most deaths during TB treatment occur in the first two months of treatment [3, 5–9]. This has been attributed to several factors including delays in health seeking, diagnosis, treatment, or a combination of them all [8, 9], delayed initiation on antiretroviral treatment in those that are HIV-positive, severe TB disease, undiagnosed drug-resistant TB (DR-TB), presence of other comorbidities, irregular or no clinical review of persons with TB receiving TB treatment and low adherence to TB treatment [8, 10, 11]. In clinically diagnosed persons, early deaths may also be due to incorrect diagnosis or very advanced disease [12].

Factors associated with increased odds of mortality among people with TB include age of 65 years and above, being retreated for TB, being HIV-positive, having unknown HIV status, clinical diagnosis, and extra-pulmonary TB (EPTB) [3, 7, 13]. Mortality has also been found to be higher among persons with EPTB, women and undernourished persons [10]. Social factors associated with high mortality among persons with TB include illiteracy and alcoholism [9, 12, 14, 15], type and location of health facility [14]. A study in Malawi found a significantly higher proportion of deaths in government-run health facilities compared to those operated by faith-based organizations [12]. Regarding the location of health facilities, a study in the Southern Province of Zambia found higher odds of death among persons with TB treated in rural settings compared to those treated in urban settings. This was attributed to limited access to TB diagnostic services for people living in rural areas [16].

The World Health Organization (WHO) defines TB death as a person infected with TB who died before starting treatment or during treatment irrespective of cause [17]. As such, most studies have used all-cause mortality as a proxy marker of mortality attributable to TB [2, 7, 10]. However, knowing the actual underlying cause of death is valuable to TB control efforts, and may help in designing effective interventions [17].

Autopsy is considered the “gold standard” for establishing the cause of death [17]. Using autopsy to determine cause of death, a study conducted in Zambia found that only 28% of the persons with TB died of a cause directly related to TB. The majority (72%) of these persons had other comorbidities [18], indicating the importance of treating comorbidities in persons with TB. This study was however limited to one hospital, probably because autopsies are expensive and hence not routinely performed in resource-limited settings like Zambia.

A more convenient method of establishing causes of death among persons with TB is a review of medical records, including death certificates [3, 17, 19]. Using this method Lin et al found that the majority (82.7%) of persons in their study died from underlying comorbidities such as malignancy, liver cirrhosis, bacterial pneumonia, renal failure, and septic shock [17]. Cardiovascular diseases have also been found to be among the common causes of death among persons with TB [3, 19].

Few studies have investigated the problem of mortality among persons with TB in Zambia. Those that have attempted to do so tend to be limited to one region of the country [3, 16, 18]. We conducted a nationwide study to determine the time to death (time taken from persons starting TB treatment up to when a death occurs); predictors of mortality; and probable causes of death.

## Materials and methods

### Study design

This was a retrospective cohort study. Medical records of persons diagnosed with drug-susceptible TB (DS-TB) in Zambia between January and December 2019 were reviewed to determine treatment outcomes. Persons with TB were followed for 12 months after initiation of treatment or until death, whichever occurred first. For persons with a treatment outcome of death, hospital files, including death certificates, where available, were reviewed to determine probable causes of death.

### Study sites

This study was conducted across three categories of Zambian hospitals: specialist hospitals (also known as tertiary referral or third-level hospitals), general hospitals (provincial or second-level hospitals), and district hospitals (first-level hospitals). At the time of data collection, Zambia had 95 hospitals: 76 first-level, 13 second-level, and six third-level facilities. These comprised 58 government hospitals, 31 faith-based (mission) hospitals, and six private hospitals [20].

Persons with presumed TB in these hospitals are entered into the presumptive TB registers, and their sputum is collected then sent to the laboratory for examination. At the laboratory, personal details including results of the sputum examination are recorded in laboratory registers. Feedback on results is provided by sending back laboratory request forms to the requesting Unit. Persons diagnosed with TB have their treatment information documented in a TB treatment register and cards. They are treated with a combination of isoniazid, rifampicin, ethambutol and pyrazinamide for a period of 6 to 12 months based on national guidelines—i.e., 6 months is the recommended period for treatment of drug-susceptible TB disease and 12 months for severe forms, such as TB meningitis [21]. Treatment outcomes are recorded in TB treatment registers and cards. For persons who die within a health facility, the probable cause of death is recorded in the person’s file and a death certificate bearing the cause of death is issued.

### Study population and sampling methods

In 2019, Zambia had 408 TB diagnostic centers. Altogether, these facilities reported 36,866 DS-TB cases [20]. For logistical reasons, this study purposefully sampled 54 hospitals that reported TB-related deaths above the national average of 4% in 2019. This included 37 first-level, 11 second-level, and 6 third-level hospitals. Together, these facilities accounted for 36% of persons with TB notified in 2019 and 48% of those reported to have died [20]. All persons diagnosed with TB between January and December 2019 in these facilities were included in the study. Persons who transferred from other districts were excluded because their treatment outcomes should have been recorded in the districts where they began treatment.

### Data collection

Between January and April 2021, this study collected secondary data on the characteristics of persons diagnosed with TB from January to December 2019, their treatment outcomes, and probable causes of death, using an electronic data extraction tool created in the District Health Information System (DHIS2).

Two facility staff captured demographic and clinical characteristics from TB treatment registers and categorized them as follows: province of registration (names of the ten provinces of Zambia); health facility ownership (public, private or mission), level of care (first, second or third level hospital); sex (man or woman); method of diagnosis (Xpert MTB/RIF or others); type of person (new or retreatment); type of TB (bacteriologically confirmed pulmonary TB (PTB), clinically diagnosed PTB, or EPTB); HIV status (positive, negative, or unknown); antiretroviral therapy (ART) treatment (on ART or not on ART); cotrimoxazole preventive therapy (CPT) (received CPT or did not receive CPT); and directly observed therapy (DOT) plan (facility-based or community-based) (Table 1). Treatment outcomes were also captured from TB treatment registers and categorized according to the Ministry of Health’s National TB Treatment Guidelines [21] (S1 File). Dates of treatment initiation and treatment outcome, including date of death, were captured from TB treatment registers and death certificates. For persons who died at home and the actual date of death was not documented, the date of last visit to the health facility was used as a proxy for the date of death.

**Table 1:** Definition of TB treatment outcomes.

Treatment outcome	Definition
Cured	A person with bacteriologically confirmed TB at the beginning of treatment, who was smear- or culture-negative in the last month of treatment and on at least one previous occasion.
Treatment completed	A person who completed TB treatment without evidence of failure, but with no record to show that sputum smear or culture results in the last month of treatment, either because tests were not done or because results were unavailable, and on at least one previous occasion was negative.
Treatment failure	A person with TB whose sputum smear or culture is positive at month five or later during treatment.
Died	A person with TB who dies for any reason before starting or during treatment.
Lost to follow-up (LTFU)	A person with TB who did not start treatment or whose treatment was interrupted for two consecutive months or more.
Not evaluated	A person with TB for whom no treatment outcome is assigned. ‘Not evaluated’ includes persons with TB who transferred out to another treatment unit in another district as well as those for whom treatment outcome is unknown to the reporting unit.

Source: Tuberculosis manual. Ministry of Health, Lusaka (2017).

To ensure data accuracy, 10 teams consisting of 3 national-level data collectors were assigned to each province. They visited a total of 54 health facilities to verify the data entered in DHIS2 by the facility-based staff. Additionally, the teams reviewed the hospital files of all deceased persons to determine the likely causes of death. Two senior medical officers in each national-level team of data collectors independently reviewed hospital files. They chronologically listed symptoms, comorbid conditions, and events preceding death. A condition or event qualified to be the probable cause of death if it had initiated the sequence of illness events leading directly to death. The cause of death recorded on the death certificate was upheld if one or both clinicians agreed. If neither of the clinicians agreed with the cause of death written on the death certificate, the two of them held a discussion to assign the probable cause of death.

### Data analysis

Data were downloaded from DHIS2 as a Microsoft Excel spreadsheet, which was imported into SPSS version 23 for cleaning and subsequently exported to Stata version 17 (17.0, StataCorp LLC, College Station, TX) for analysis. Data were summarized as frequencies and percentages for categorical variables (e.g., clinical and demographic characteristics, treatment outcomes, and causes of death) and medians for continuous variables (e.g., age and time to death). Time to death was calculated by subtracting the date of treatment initiation from the date of treatment outcome and divided by 30 to obtain the total months of follow-up for all persons. We summarized survival probabilities in life tables. We presented Kaplan-Meier survival curves with associated log-rank test p-values.

Multiple imputation methods by chain equations (20 times) were used to handle missing data on risk factors after an assessment of the pattern of missing values that were missing at random (MAR) [22]. Imputed variables included age, sex, type of TB, type of person, HIV status and DOT plan. Cox regression models were fitted to estimate predictors of mortality, and we reported both unadjusted and adjusted hazard ratios (uHR and aHR). Schoenfeld’s global test p-value greater than 0.05 indicated that the proportional hazard regression assumption was met.

The outcome variable was binary defined as died or did not die (“did not die” included all the following categories: cured, treatment completed, treatment failure, and lost to follow-up). All persons with TB treatment outcomes other than death (our event of interest) were right censored at the end of the follow-up period (12 months). Variables with a p-value <0.20 on bivariate analysis were included in the multivariate model. Variables with a p-value <0.05 at multivariate analysis were considered statistically significant mortality predictors among persons with TB. Multicollinearity among predictor variables was tested using the variance inflation factor (VIF) method. Correlation coefficients of less than 0.8 and VIF of less than 10 indicated no multicollinearity among predictor variables. We conducted a sensitivity analysis to assess the robustness of our results by running an identical multivariate model that recorded all persons who were LTFU and those whose treatment outcome was not evaluated as having died.

At analysis stage, the probable causes of death recorded on death certificates as well as those assigned by reviewing physicians were grouped under thirteen broad categories: PTB, disseminated TB, sepsis, end-organ damage, anemia, TB meningitis, superadded pneumonia, adrenal insufficiency, aspiration pneumonia, sequelae of TB, other central nervous system (CNS) infections, and other causes (e.g. malignancy, malaria, trauma).

### Ethics statement

This study was approved by the University of Zambia Biomedical Research Ethics Committee (Ref: 1216–2020). The requirement for consent was waived because our study relied on secondary data from registers and files. The National Health Research Authority granted permission to conduct the study (Ref: NHRA000014/30/10/2020).

The reporting of this study conforms to the Strengthening the Reporting of Observational Studies in Epidemiology (STROBE) guidelines (S2 File).

## Results

### Description of the cohort

**Fig 1:**
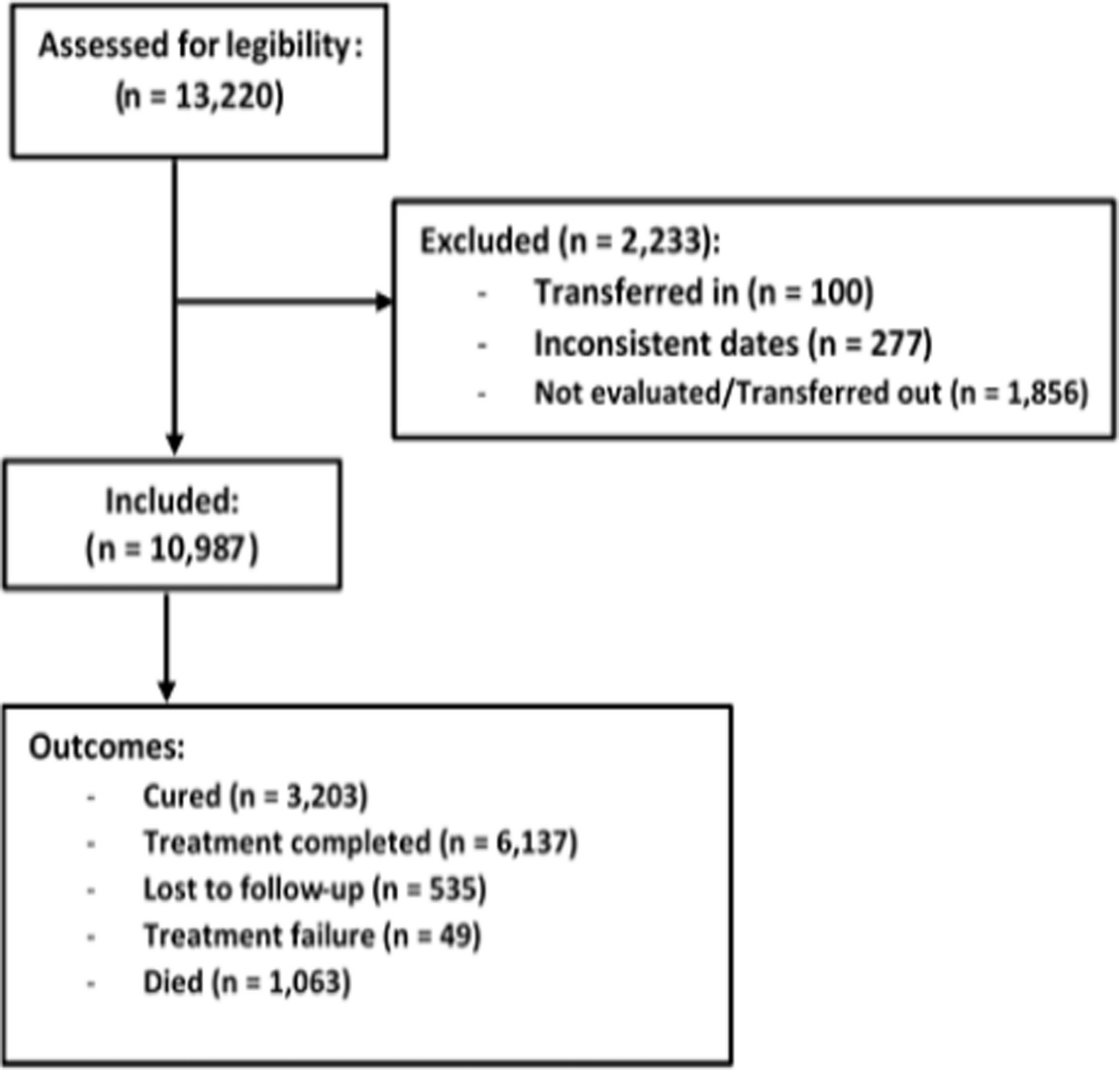
Flow chart for persons included in the study on mortality among persons on tuberculosis treatment in Zambian hospitals, 2019.

We abstracted records for 13,220 persons with DS-TB from registers at the 54 hospitals and excluded from the analysis one hundred persons who were transferred into these hospitals. An additional 277 persons were excluded because of inconsistent start and end dates. We also excluded 1,856 persons whose treatment outcomes were not evaluated or were transferred out. The final analysis was on 10,987 persons (Fig 1).

The majority, 6,761 (61.5%) of persons were male. The median age was 36 years (IQR: 27–46 years). The largest proportion 4,540 (41.3%) of the persons were from Lusaka province, and government hospitals (86.5%). First-level hospitals accounted for more than half, 5,685 (51.1%) of the persons, and third-level and second-level hospitals accounted for 2,662 (24.2%) and 2,640 (24.0%), respectively (Table 2).

**Table 2:** Demographic and clinical characteristics of persons with TB Zambian hospitals (2019), n = 10,987.

Category	Number of persons who died (%), n = 1,063	Number of persons who did not die (%), n = 9,924	Total(%), n = 10,987
Province			
Central	35 (2.9)	159 (1.6)	194 (1.6)
Copperbelt	115(10.8)	1,095 (11.0)	1,210 (11.0)
Eastern	110 (10.4)	629(6.3)	739 (6.7)
Luapula	37 (3.5)	606 (6.1)	643 (5.9)
Lusaka	424 (39.9)	4,116 (41.5)	4,540 (41.3)
Muchinga	53 (5.0)	415 (4.2)	468 (4.3)
North-Western	71 (6.7)	994 (10.0)	1,065 (9.7)
Northern	21 (2.0)	658 (6.6)	679 (6.2)
Southern	135 (12.7)	796 (8.0)	931 (8.5)
Western	62 (5.8)	456 (4.6)	518 (4.7)
Health facility ownership			
Government	879 (82.7)	8,628 (86.9)	9,507 (86.5)
Private	3 (0.3)	29 (0.3)	32 (0.3)
Mission	181 (17.0)	1,267 (12.8)	1,448 (13.2)
Level of care			
First-level hospital	477 (44.9)	5,208 (52.5)	5685 (51.7)
Second-level hospital	219 (20.6)	2,421 (24.4)	2,640 (24.0)
Third-level hospital	367 (34.5)	2,295 (23.1)	2,662 (24.2)
Age			
0–4	30 (2.8)	448 (4.5)	478 (4.4)
5–14	28 (2.6)	503 (5.1)	531 (4.8)
15–24	79 (7.4)	1,148 (11.6)	1,227 (11.2)
25–34	219 (20.6)	2,407 (24.3)	2,626 (23.9)
35–44	280(26.3)	2,659 (26.8)	2,939 (26.8)
45–54	190 (17.9)	1,466 (14.8)	1,656 (15.1)
55–64	107 (10.1)	709 (7.1)	816 (7.4)
65 and above	128 (12.0)	563 (5.7)	691 (6.3)
Missing	2 (0.2)	21 (0.2)	23 (0.2)
Sex			
Female	429 (40.4)	3,789 (38.2)	4,218 (38.4)
Male	634 (59.6)	6,127 (61.7)	6761 (61.5)
Missing	0	8 (0.1)	8 (0.1)
Type of TB			
Bacteriologically confirmed PTB	268 (25.2)	4,032 (40.6)	4,300 (39.1)
Clinically diagnosed PTB	524 (49.3)	4,368 (44.0)	4,892 (44.5)
EPTB	263 (24.7)	1,483 (14.9)	1,746 (15.9)
Missing	8 (0.8)	41 (0.4)	49 (0.5)
Type of person			
New	888(83.5)	8,480 (85.6)	9,368 (85.3)
Retreatment	175 (16.5)	1,441 (14.5)	1,616 (14.7)
Missing	0 (0.0)	3 (0.0)	3 (0.0)
Diagnosed using Xpert MTB/RIF			
Yes	240 (22.6)	3,648 (36.8)	3,888 (35.4)
No	823 (77.4)	6,276 (63.2)	7,099 (64.6)
HIV status			
Negative	369(34.7)	5,039 (50.8)	5408 (49.2)
Positive	671 (63.1)	4,745 (47.8)	5,416 (49.3)
Unknown/missing	23 (2.2)	140 (1.4)	163 (1.5)
DOT plan			
Community-based	674 (63.4)	7,776 (78.4)	8,450 (76.9)
Facility-based	155 (14.6)	1,689 (17.0)	1,844 (16.8)
Missing	234 (22.0)	459 (4.6)	693 (6.3)
On ART (n = 5,416)	n = 671	n = 4,745	n = 5,416
Yes	594(88.5)	4,491 (94.7)	5,085 (93.9)
No	43 (6.4)	119 (2.5)	162 (3.0)
Missing	34 (5.0)	135 (2.9)	169 (3.1)
On CPT (n = 5,416)	n = 671	n = 4,745	n = 5,416
Yes	425 (63.3)	3,399 (71.6)	3,824 (70.6)
No	105 (15.7)	295 (6.2)	400 (7.4)
Missing	141 (21.0)	1,051 (22.6)	1,192 (22.0)

ART, antiretroviral therapy; CPT, cotrimoxazole presumptive therapy; DOT, directly observed therapy; TB, tuberculosis; EPTB, extra-pulmonary tuberculosis; HIV, human immunodeficiency virus; PTB, pulmonary tuberculosis; MTB RIF, mycobacterium tuberculosis and resistance to rifampin

The majority, 9,192 (83.6%) persons included in the study had PTB. Of these, 4,300 (46.8%) had bacteriologically confirmed PTB, while 4,892 (53.2%) had clinically diagnosed PTB, and the remaining 1,746 (15.9%) had EPTB. Most (85.3%) of the persons with TB were newly diagnosed. Retreatments (relapses, treatment after failure, treatment after loss to follow-up and others) accounted for 1,616 (14.7%). Only 3,888 (35.4%) of the persons were diagnosed using Xpert MTB/RIF versus microscopy, and X-ray (7,099 or 64.6%) (Table 2).

Out of the total, 10,987 persons with TB (49.3%) were HIV positive, while 5,408 (49.2%) tested negative for HIV and 163 (1.5%) has unknown HIV status. Among those who were HIV positive, 5,085 (93.9%) were receiving antiretroviral therapy (ART), and 3,824 (70.6%) were on cotrimoxazole preventive therapy (CPT). The majority of persons, (76.9%), were on a community-based DOT plan, which involved being observed by a relative or a community volunteer. On the other hand, 1,844 (16.8%) were on a facility-based DOT plan (Table 2).

### Treatment outcomes for persons with TB

Of the 10,987 persons with TB who were on treatment, 3,203 (29.2%) were cured, 6,137 (55.8%) completed treatment, 535 (4.9%) were lost to follow-up, and 1,063 (9.7%) died. Treatment failed in 49 (0.5%) persons. (Table 3). The overall incidence rate was 16.9 deaths per 1,000 person-months of observation (CI: 15.9–17.9). The median age at time of death was 40 years (IQR: 31–52 years).

**Table 3:** Treatment outcomes for persons with TB in Zambian hospitals (2019), n = 10,987.

Treatment outcome	Number of persons with TB	Total (%)	Person-time (in months)
Cured	3,203	29.2	19,996.1
Treatment completed	6,137	55.8	39,001.9
Lost to follow-up	535	4.9	2,261.9
Treatment failure	49	0.5	248.1
Died	1,063	9.7	1496.5
Total	10,987	100.0	63,004.5

### Time to death

**Fig 2:**
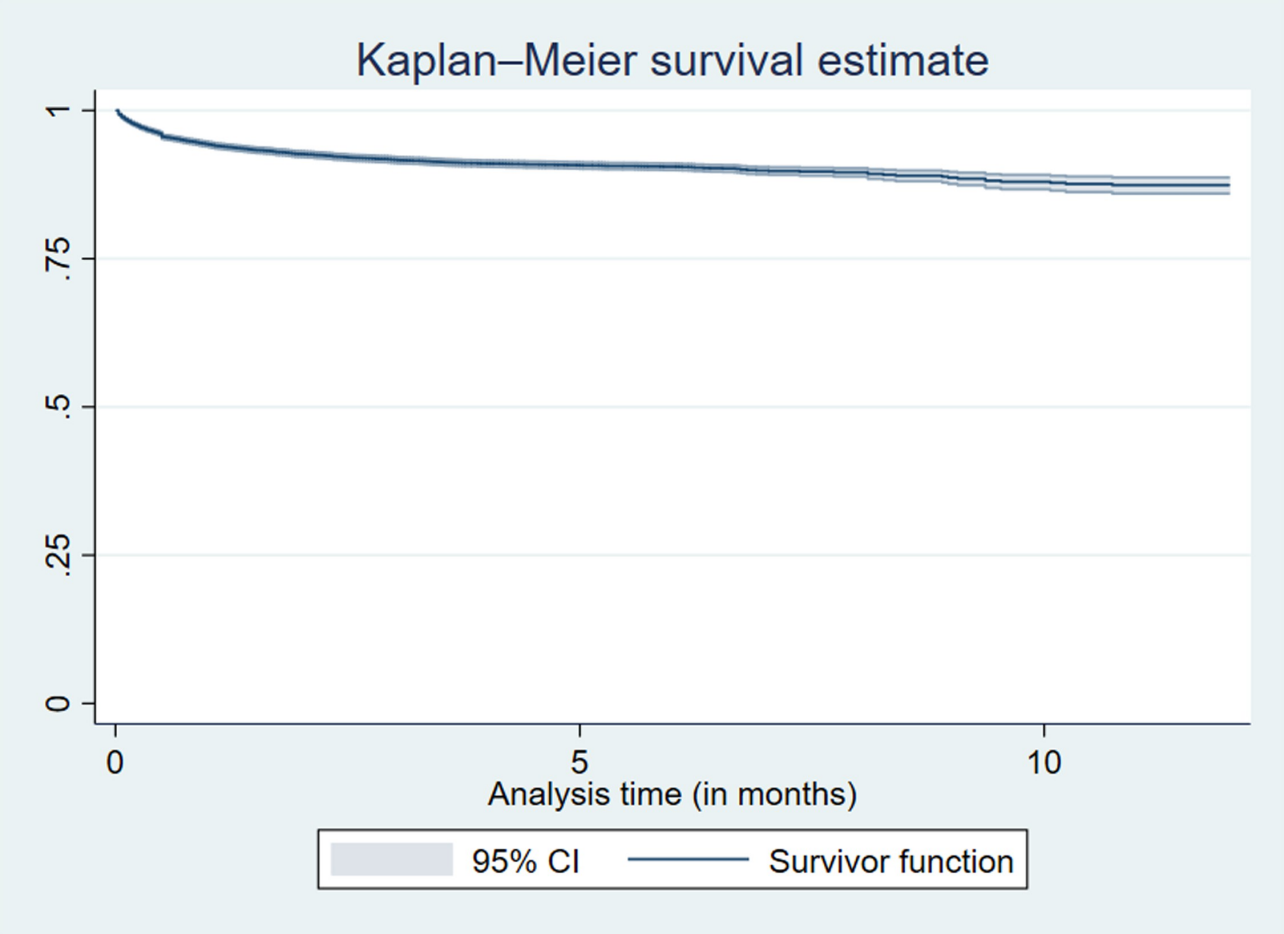
Kaplan-Meier survival estimate for persons on tuberculosis treatment in Zambian hospitals, 2019.

Of the 1,063 deaths recorded in the 12 months of observation, more than half (58.1%) of the deaths occurred in the first month of TB treatment, and 75.1% in the first two months (Table 4) (Fig 2). The median time to death was 21 days (IQR: 6–57). The survival probabilities differed significantly by age, facility type, HIV status, method of diagnosis, and type of TB (S1 Fig).

**Table 4:** Time to death for persons with TB in Zambian hospitals (2019), n = 10,987.

Interval (in months)	Total persons	Deaths (%)	Censored	Cumulative survival probabilities (%)	95% CI
0 to 1	10,987	618 (58.1)	145	94.3	93.3–94.8
1 to 2	10,224	181 (17.0)	77	92.7	92.2–93.1
2 to 3	9,966	104 (9.8)	70	91.7	91.2–92.2
3 to 4	9,792	69 (6.5)	81	91.70	90.5–91.6
4 to 5	9,642	31 (2.9)	149	90.8	90.2–91.3
5 to 6	9,462	24 (2.3)	1,496	90.5	89.9–91.0
6 to 7	7,942	20 (1.9)	6,526	90.1	89.5–90.7
7 to 8	1,396	3 (0.3)	719	89.9	89.2–90.5
8 to 9	674	6 (0.6)	138	89.0	88.0–89.9
9 to 10	530	4 (0.4)	42	88.3	87.0–89.4
10 to 11	484	3 (0.3)	28	87.7	86.3–89.0
11 to 12	453	0 (0.0)	453	87.7	86.3–89.0

CI, confidence interval

### Predictors of mortality

The mortality hazard in persons aged 55–64 years was 1.5 times the hazard in those aged 35–44 years (aHR: 1.5; CI: 1.2–1.8). The mortality hazard in persons aged above 65 years was 2.4 times the hazard in those aged 35–44 years (aHR: 2.4; CI: 1.9–3.0). Generally, the likelihood of death increased with age. Compared to Lusaka Province, persons treated in all the provinces (except for Copperbelt, Northern, North-Western and Luapula provinces) were more likely to die (aHR > 1 and p-values <0.05). Persons treated in mission hospitals had 1.6 times the mortality hazard as those treated in government hospitals (aHR: 1.6; CI: 1.3–2.0). Compared to those treated at first-level hospitals, persons treated at third-level hospitals were twice as likely to die (aHR: 2.1; CI 1.7–2.5). Persons with EPTB had 1.7 times the mortality hazard compared to those with bacteriologically confirmed PTB (aHR: 1.7; CI: 1.4–2.2). Mortality hazard for persons diagnosed using methods other than Xpert MTB/RIF increased by 30% (aHR: 1.3; CI: 1.1–1.7). Persons with TB who were co-infected with HIV had 1.8 times the mortality hazard as those who were HIV-negative (aHR: 1.8; CI: 1.6–2.1) (Table 5). In a sub-analysis of persons who were co-infected with HIV, those who were not on ART were more than twice as likely to die compared to those who were on ART (aHR: 2.4; CI 1.8–3.3) (S1 Table). A sensitivity analysis in which all persons LTFU were re-coded as having died also showed that province of treatment, type of facility, age, sex, method of diagnosis, and HIV status were significant predictors of mortality among persons with TB (S2 Table).

**Table 5:** Predictors of mortality among persons with TB Zambian hospitals (2019), n = 10,987.

Category	uHR (95% CI)	p-value	aHR (95% CI)	p-value
	Univariate		Multivariate	
Province				
Central	2.1(1.5–2.9)		3.8 (2.6–5.5)	<0.001
Copperbelt	1.0 (0.8–1.3)		0.8 (0.7–1.0)	0.100
Eastern	1.6 (1.3–2.0)		2.5 (1.9–3.2)	<0.001
Luapula	0.6 (0.4–0.8)		0.8 (0.5–1.2)	0.261
Lusaka	1		1	
Muchinga	1.2 (0.9–1.7)		2.3 (1.6–3.1)	<0.001
North-Western	0.7 (0.6–0.9)		1.0 (0.7–1.3)	0.849
Northern	0.3 (0.2–0.5)		0.6 (0.4–1.0)	0.067
Southern	1.6 (1.4–2.0)		2.0 (1.5–2.6)	<0.001
Western	1.3 (1.0–1.7)	<0.001	2.0 (1.5–2.7)	<0.001
Health facility ownership				
Government	1		1	
Private	1.0 (0.3–3.0)		1.7 (0.5–5.4)	0.373
Mission	1.4 (1.2–1.6)	0.001	1.6(1.3–2.0)	<0.001
Level of care				
First level Hospital	1		1	
Second level Hospital	1.0 (0.8–1.2)		0.9 (0.8–1.1)	0.456
Third level Hospital	1.6 (1.4–1.9)	<0.001	2.1 (1.7–2.5)	<0.001
Age				
0–4	0.6 (0.4–0.9)		0.9 (0.6–1.3)	0.484
5–14	0.5 (0.4–0.8)		0.6 (0.4–0.9)	0.014
15–24	0.7 (0.5–0.9)		0.8 (0.6–1.0)	0.104
25–34	0.9 (0.7–1.0)		1.0 (0.8–1.2)	0.757
35–44	1		1	
45–54	1.2 (1.0–1.5)		1.2 (1.0–1.5)	0.037
55–64	1.4 (1.1–1.7)		1.5 (1.2–1.8)	0.001
65 and above	2.0 (1.7–2.5)	<0.001	2.4 (1.9–3.0)	<0.001
Sex				
Male	1		1	
Female	1.1 (1.0–1.2)	0.195	1.0 (0.9–1.1)	0.571
Type of TB				
PTB (confirmed)	1		1	
PTB (Clinical)	1.7 (1.5–2.0)		1.2 (1.0–1.5)	0.067
EPTB	2.5 (2.1–2.9)	<0.000	1.7 (1.4–2.2)	<0.001
Type of person				
New	1		1	
Retreatment	1.1 (1.0–1.3)	0.098	1.0 (0.8–1.2)	0.973
Diagnosed using Xpert MTB/RIF				
Yes	1		1	
No	1.9 (1.7–2.2)	<0.001	1.3 (1.1–1.7)	0.012
HIV status				
Negative	1		1	
Positive	1.8 (1.6–2.1)	<0.001	1.8 (1.6–2.1)	<0.001
DOT plan				
Community-based	1		-	-
Facility-based	1.0 (0.9–1.2)	0.754	-	-

CI, confidence interval; DOT, directly observed therapy; TB, tuberculosis; EPTB, extra-pulmonary tuberculosis; HIV, human immunodeficiency virus; PTB, pulmonary tuberculosis; MTB RIF, mycobacterium tuberculosis and resistance to rifampin; uHR, crude hazard ratio; aHR: adjusted hazard ratio

### Probable causes of death among persons with TB

Out of 1,063 persons with TB who died, death certificates were available for 207 (19.5%) and medical files were available for 499 (46.9%). Findings from the review of medical files agreed with records captured from death certificates that the top four probable causes of death among persons with TB notified in Zambian hospitals included: TB IRIS/disseminated TB, PTB, septic shock and end-organ damage. Anaemia and TB meningitis were the other common probable causes of death (Table 6). Among the 67 persons who died of end-organ damage, the commonest probable cause of death was heart failure, which was reported in 30 (45%) of the persons (S2 Fig). In a sub-analysis that only considered persons living with HIV, TB IRIS/disseminated, sepsis/septic shock, end organ damage and aneamia were the top four probable causes of death (S3 Table). On the other hand, sepsis, TB IRIS/disseminated, PTB and end-organ damage were among the top four probable causes of death among those not living with HIV (S4 Table).

**Table 6:** Probable causes of death among persons with TB Zambian hospitals (2019).

Probable cause of death on certificates	#	%	Assigned probable causes of death	#	%
(n = 207)			(n = 499)		
Disseminated TB	44	21.3	Sepsis	94	18.8
PTB	42	20.3	Disseminated TB	89	17.8
Sepsis/septic shock	23	11.1	End organ damage	67	13.4
End organ damage	20	9.7	PTB	57	11.4
TB meningitis	20	9.7	Anaemia	48	9.6
Superadded pneumonia	16	7.7	TB meningitis	39	7.8
Anaemia	15	7.3	Other causes (malignancy, malaria, trauma)	39	7.8
Sequelae of TB	9	4.4	Superadded pneumonia	26	5.2
Other CNS infections	7	3.4	Adrenal insufficiency	20	4.0
Other (causes, malignancy, malaria, trauma)	6	2.9	Other CNS infections	16	3.2
Aspiration pneumonia	3	1.5	Aspiration pneumonia	3	0.6
Adrenal insufficiency	2	1.0	Sequelae of TB	1	0.2
**Total**	**207**	**100**	**Total**	**499**	**100**

CNS, central nervous system; TB, tuberculosis; EPTB, extra-pulmonary tuberculosis; PTB, pulmonary tuberculosis

## Discussion

A total of 1,063 persons included in this study died while on TB treatment, translating into a mortality rate of 9.7%, which was lower than the 16.3% found in South Africa [13], but higher than the 3.4% reported in Tanzania [2]. Differences in TB mortality rates among countries could be due to varying degrees in the prevalence of comorbidities such as HIV, anaemia, diabetes mellitus, malignancies and renal failure, among other reasons [2]. In Zambia, HIV continues to be the main driver of TB disease. Over half of (50.1%) persons receiving TB treatment were co-infected with HIV, hence, optimal HIV management in persons co-infected with HIV and TB coupled with treatment adherence support, could help reduce mortality.

Our study found that death predominantly occurred in the first 2 months of TB treatment, which is in agreement with earlier studies conducted in Malawi, Ethiopia and Zimbabwe [6, 7, 12]. Studies have identified risk factors for early death during the course of TB treatment that include inadequate access to services, delays in TB diagnosis and/or initiation of anti-TB and antiretroviral treatment, severe disease, undiagnosed DR-TB, presence of other comorbidities, and low adherence to anti-TB drugs [6, 8, 10].

Our study did not capture the timing of ART and anti-TB treatment initiation to rule out delays in their initiation. However, delays in TB diagnosis in Zambia were confirmed by the 2014 National TB Prevalence Survey which showed that only 35% of presumptive TB cases sought care for their symptoms [23]. Among those that sought care, diagnosis was missed in 50% of them, indicating a low index of suspicion for TB among healthcare workers [23]. Delayed diagnosis could therefore be among the reasons for high mortality in the initial phase of TB treatment reported by this study. Community sensitization on the importance of seeking care early, taking TB diagnostic facilities closer to where people live, and capacity building for healthcare workers may therefore help reduce “early” deaths among persons on TB treatment.

Our study also found that compared to those treated in Lusaka Province (the most urbanized province in Zambia), persons treated in Muchinga, Eastern, Western, Southern and Central Provinces were more likely to die. This was despite Lusaka Province having the highest proportion (58.2%) of persons with TB who were co-infected with HIV, a known risk factor for TB-related death. A previous study conducted in Tanzania attributed this to differences in socioeconomic status and variation in the death risk across geographical regions [24]. Differences in socioeconomic status across the ten provinces of Zambia could explain our findings. The five provinces that recorded significantly higher risk of TB mortality compared to Lusaka, had more than twice higher poverty levels than Lusaka according to a 2022 poverty assessment for Zambia [25]. Therefore, poor socio-economic status may explain the higher risk of mortality in Muchinga, Eastern, Western, Southern and Central Provinces. Further research may however be required to understand the relatively lower risk of TB mortality in Northern and Luapula Provinces—regions with the third and fourth highest levels of poverty, respectively [25].

Our study also found that persons with TB who received treatment in mission hospitals were more likely to die compared to those treated in government hospitals. A possible explanation could be that mission hospitals are located in remote rural areas where it is a challenge for the population to navigate long distances to the nearest hospital compared to government hospitals. Further operational research is required to understand this finding.

Mortality in this study was also disproportionately higher in third-level hospitals. This could be linked to the delayed referral to a higher level of care. In Zambia, more persons with TB present at first and second-level hospitals than directly at third-level. Third-level are mainly referral hospitals. Hence, the relatively higher mortality at Third-level hospitals could suggest late referral and/or advanced disease. Emphasis on early referral of complicated TB cases and improving diagnostic and treatment capabilities at lower levels of care may help reduce TB related mortalities at higher levels of care.

In this study, persons co-infected with HIV and TB were more likely to die than those that were HIV-negative. This is an expected finding as the relationship between TB-HIV comorbidity and death is well-established in the literature [3, 13, 24]. This was notwithstanding that over 90% of the persons living with HIV were taking ART. We could not explicitly associate the high mortality linked to HIV with antiretroviral treatment failure as we did not collect any information on the viral loads, timing of ART initiation and period of taking ART.

Further, our results showed that a significant proportion of the persons who died had acute end-organ damage, which in some circumstances involved multiple organs. To the best of our knowledge, the end-organ damage could be linked to disseminated TB disease and complications of the disease. Our findings support those from a previous study conducted in Zambia, which showed that over 30% of persons that died while on TB treatment, died of non-TB comorbidities that included anemia, cardiac failure and *Pneumocystis jiroveci pneumonia* [3]. Although both the current and the previous study are limited by the absence of autopsy reports, a comprehensive screening and treatment of comorbidities can help prevent death among persons receiving TB treatment.

The greatest strength of our study was its large sample size drawn from all the 10 provinces of Zambia, giving a nationally representative picture. Besides TB registers, medical files were also reviewed, including death certificates, for those who died. This enabled us to collect data on comorbidities and probable causes of death that are not routinely captured in TB registers.

This study had several limitations. Firstly, we only included sites that reported TB mortality above the national average, which limits the generalizability of our findings. Secondly, a high number of records were missing data on some variables (21.7%). We reduced the effect of this limitation by using multiple imputations to handle missing data on covariates. We could not ascertain the status (died vs did not die) for persons who were lost-to-follow-up. In our analysis of predictors of mortality, we treated these persons as not having died. We also excluded from our analysis persons whose treatment outcome was not evaluated/missing. These factors may have reduced the probability of observing death in our model. A sensitivity analysis in which all persons who were LTFU were re-coded as having died did not however change our conclusions. Thirdly, the lack of information on immunity status (CD4 counts) and viral loads for persons living with HIV meant our study could not rule out failing ART as one of the predictors of mortality in this subgroup. Similarly, almost half of the cohort had clinically diagnosed TB with no microbiological test nor MTB resistance test. It is possible that some of the deaths were due to drug-resistant TB, or other undiagnosed ailments.

We did not perform autopsy, which is considered the “gold standard” for establishing cause of death [16]. We relied on information from medical files and death certificates, and the specific cause of death might not have been accurate. Experienced physicians were trained to collect data on probable causes of death, which may have helped minimize the impact of this limitation on the findings of our study. Furthermore, we could not find medical files for 60% of the persons who died, Thus the probable causes of death reported by our study may not be an accurate representation of causes of death among persons with TB. Finally, data collection on persons who did not die was limited to variables that are routinely captured in TB treatment registers. As a result, we could not make inferences on how other clinical (time to TB diagnosis, and adherence to TB treatment) and socioeconomic factors (occupation, history of smoking, alcohol intake, nutritional status and comorbidities such as diabetes and hypertension) influence mortality among Persons with TB.

## Conclusions

This is the first-ever comprehensive countrywide TB mortality study in Zambia. The study demonstrates that TB mortality is high in Zambia with the majority of persons dying within the first two months of treatment initiation. Risk factors for dying included being: elderly (aged 55 years and above), treated in a province outside Lusaka (Muchinga, Eastern, Western, Southern and Central), treated at third-level hospitals, treated in mission hospital and diagnosed with methods other than Xpert MTB/RIF. Others included having EPTB and living with HIV. The disproportionate mortality rates across different settings and person categories reveal the importance of targeted (differentiated) care for persons with TB. Our results also point towards the necessity of integrated TB care that include comprehensive HIV care, and the management of TB IRIS, septic shock, end-organ damage, anemia and TB meningitis, which were found to be among the common probable causes of death. Further operational research is imperative to address the nuances of TB treatment outcomes and to develop targeted interventions that can significantly reduce mortality rates among persons receiving TB treatment in Zambia.

## Supporting information

S1 File Detailed definition variables used in the study on mortality among persons receiving tuberculosis treatment in Zambian hospitals. (DOCX)

S2 File STROBE statement—checklist of items that should be included in reports of observational studies. (DOCX)

S1 Table Predictors of mortality among persons with TB Zambian hospitals: A sub-analysis of people living with HIV (2019), n = 5,416. (DOCX)

S2 Table Sensitivity analysis of predictors of mortality among persons with TB s in Zambian hospitals, recoding all persons LTFU as having died (2019), n = 10,987. (DOCX)

S3 Table Probable causes of death among persons with TB Zambian hospitals: A sub-analysis of people living with HIV (2019). (DOCX)

S4 Table Probable causes of death among persons with TB Zambian hospitals: A sub-analysis of people not living with HIV (2019). (DOCX)

S1 Fig Kaplan-Meier survival estimate for persons on TB treatment in Zambia in 2019 by sex, age, facility type, HIV status, method of diagnosis and type of TB (2019), n = 10,987. (DOCX)

S2 Fig Comorbid condition or end organ damage as a cause of death among persons with TB in Zambia (2019), n = 499. (DOCX)
