# Supplementary material for: Mortality among persons with tuberculosis in Zambian hospitals: A retrospective cohort study
Source: PLOS Glob Public Health. 2024 Jun 17;4(6):e0003329. doi: 10.1371/journal.pgph.0003329 (PMC11182540; doi:10.1371/journal.pgph.0003329)
Supplement: S1 Table — (DOCX) [file pgph.0003329.s003.docx]

**S1 Table.**  **Predictors of mortality among persons with TB Zambian hospitals: A sub-analysis of people living with HIV (2019), n= 5,416**

| **Category** | **uHR (95% CI)** | **p-value** | **aHR (95% CI)** | **p-value** |
| --- | --- | --- | --- | --- |
|  | **Univariate** |  | **Multivariate** |  |
| Province |  |  |  |  |
| Central | 1.7 (1.1-2.7) |  | 3.5 (2.2-5.6) | <0.001 |
| Copperbelt | 1.0 (0.8-1.3) |  | 0.9 (0.7-1.2) | 0.100 |
| Eastern | 1.7 (1.3-2.2) |  | 2.6 (1.8-3.7) | <0.001 |
| Luapula | 0.6 (0.4-1.0) |  | 0.8 (0.4-1.3) | 0.261 |
| Lusaka | 1 |  | 1 |  |
| Muchinga | 1.4 (1.0-2.2) |  | 2.7 (1.7-4.2) | <0.001 |
| North-Western | 0.6 (0.4-0.9) |  | 0.8 (0.5-1.3) | 0.849 |
| Northern | 0.4 (0.2-0.8) |  | 0.7 (0.3-1.4) | 0.067 |
| Southern | 1.5 (1.1-1.9) |  | 1.9 (1.4-2.7) | <0.001 |
| Western | 1.6 (1.1-2.3) | <0.001 | 2.6 (1.7-3.9) | <0.001 |
| Health facility ownership |  |  |  |  |
| Government | 1 |  | 1 |  |
| Private | 0.8 (0.2-3.3) |  | 1.5 (0.4-6.3) | 0.373 |
| Mission | 1.4 (1.1-1.7) | 0.001 | 1.8 (1.3-2.4) | <0.001 |
| Level of care |  |  |  |  |
| First-level hospital | 1 |  | 1 |  |
| Second-level hospital | 1.2 (0.9-1.4) |  | 1.0 (0.8-1.4) | 0.456 |
| Third-level hospital | 1.6 (1.4-1.9) | <0.001 | 2.2 (1.7-2.8) | <0.001 |
| Age |  |  |  |  |
| 0 - 4 | 0.8 (0.4-1.5) |  | 0.8 (0.4-1.5) | 0.484 |
| 5 - 14 | 0.7 (0.4-1.2) |  | 0.7 (0.4-1.2) | 0.014 |
| 15 - 24 | 0.9 (0.6-1.2) |  | 0.9 (0.6-1.2) | 0.104 |
| 25 - 34 | 1.1 (0.9-1.3) |  | 1.1 (0.9-1.3) | 0.757 |
| 35 - 44 | 1 |  | 1 |  |
| 45 - 54 | 1.3 (1.0-1.6) |  | 1.3 (1.0-1.6) | 0.037 |
| 55 - 64 | 1.5 (1.1-2.0) |  | 1.4 (1.0-1.8) | 0.001 |
| 65 and above | 2.0 (1.4-3.1) | <0.001 | 1.8 (1.2-2.7) | <0.001 |
| Sex |  |  |  |  |
| Male | 1 |  | 1 |  |
| Female | 1.0 (0.9-1.2) | 0.616 | 1.0 (0.8-1.1) | 0.571 |
| Type of TB |  |  |  |  |
| PTB (confirmed) | 1 |  | 1 |  |
| PTB (Clinical) | 1.4 (1.2-1.7) |  | 1.1 (0.8-1.4) | 0.067 |
| EPTB | 2.2 (1.8-2.8) | <0.000 | 1.5 (1.2-2.1) | <0.001 |
| Type of person |  |  |  |  |
| New | 1 |  | 1 |  |
| Retreatment | 0.9 (0.8-1.2) | 0.098 | 1.0 (0.8-1.2) | 0.973 |
| Diagnosed using Xpert MTB/RIF |  |  |  |  |
| Yes | 1 |  | 1 |  |
| No | 1.7 (1.4-2.0) | <0.001 | 1.4 (1.1-1.8) | 0.012 |
| On ART |  |  |  |  |
| Yes | 1 |  | 1 |  |
| No | 2.5 (1.8-3.4) | <0.001 | 2.4 (1.8-3.3) | <0.001 |
| DOT plan |  |  |  |  |
| Community-based | 1 |  | - | - |
| Facility-based | 1.1 (0.9-1.3) | 0.876 | - | - |

CI, confidence interval; DOT, directly observed therapy; TB, tuberculosis; EPTB, extra-pulmonary tuberculosis; HIV, human immunodeficiency virus; PTB, pulmonary tuberculosis; MTB RIF, mycobacterium tuberculosis and resistance to rifampin; uHR, crude hazard ratio; aHR: adjusted hazard ratio
