# Supplementary material for: Mortality among persons with tuberculosis in Zambian hospitals: A retrospective cohort study
Source: PLOS Glob Public Health. 2024 Jun 17;4(6):e0003329. doi: 10.1371/journal.pgph.0003329 (PMC11182540; doi:10.1371/journal.pgph.0003329)
Supplement: S2 Table — (DOCX) [file pgph.0003329.s004.docx]

**S1 Table. Sensitivity analysis of predictors of mortality among persons receiving TB treatment in Zambian hospitals, recoding all persons LTFU as having died (2019), n=10,978**

| **Category** | **uHR (95% CI)** | **p-value** | **aHR (95% CI)** | **p-value** |
| --- | --- | --- | --- | --- |
|  | **Univariate** |  | **Multivariate** |  |
| Province |  |  |  |  |
| Central | 1.9 (1.4-2.6) |  | 3.2 (2.3-4.5) | <0.001 |
| Copperbelt | 0.9 (0.7-1.1) |  | 0.7 (0.6-0.9) | 0.004 |
| Eastern | 1.6 (1.3-1.9) |  | 2.2 (1.8-2.8) | <0.001 |
| Luapula | 0.7 (0.5-0.9) |  | 0.9 (0.6-1.2) | 0.383 |
| Lusaka | 1 |  | 1 |  |
| Muchinga | 1.2 (0.9-1.5) |  | 1.9 (1.5-2.6) | <0.001 |
| North-Western | 1.8 (1.5-2.1) |  | 2.3 (1.9-2.8) | <0.001 |
| Northern | 0.6 (0.4-0.8) |  | 1.0 (0.7-1.4) | 0.838 |
| Southern | 2.0 (1.7-2.3) |  | 2.4 (1.9-3.0) | <0.001 |
| Western | 1.4 (1.2-1.8) | <0.001 | 2.1 (1.7-2.7) | <0.001 |
| Health facility ownership |  |  |  |  |
| Government | 1 |  | 1 |  |
| Private | 0.7 (0.2-2.2) |  | 1.4 (0.4-4.3) | 0.599 |
| Mission | 1.7 (1.5-1.9) | <0.001 | 1.5 (1.3-1.8) | <0.001 |
| Level of care |  |  |  |  |
| First-level hospital | 1 |  | 1 |  |
| Second-level hospital | 0.9 (0.8-1.1) |  | 0.9 (0.7-1.0) | 0.079 |
| Third-level hospital | 1.2 (1.1-1.4) | 0.171 | 1.9 (1.6-2.2) | <0.001 |
| Age |  |  |  |  |
| 0 - 4 | 0.7 (0.6-1.0) |  | 0.8 (0.6-1.1) | 0.285 |
| 5 - 14 | 0.8 (0.6-1.0) |  | 0.8 (0.6-1.0) | 0.052 |
| 15 - 24 | 0.8 (0.7-1.0) |  | 0.9 (0.7-1.1) | 0.318 |
| 25 - 34 | 1.0 (0.9-1.2) |  | 1.1 (0.9-1.2) | 0.287 |
| 35 - 44 | 1 |  | 1 |  |
| 45 - 54 | 1.2 (1.0-1.4) |  | 1.2 (1.0-1.4.) | 0.041 |
| 55 - 64 | 1.5 (1.2-1.8) |  | 1.4 (1.2-1.7) | <0.001 |
| 65 and above | 1.9 (1.6-2.2) | <0.001 | 1.9 (1.6-2.4) | <0.001 |
| Sex |  |  |  |  |
| Male | 1 |  | 1 |  |
| Female | 1.0 (0.9-1.1) | 0.579 | 0.9 (0.8-1.0) | 0.026 |
| Type of TB |  |  |  |  |
| PTB (confirmed) | 1 |  | 1 |  |
| PTB (Clinical) | 1.5 (1.3-1.7) |  | 1.0 (0.9-1.3) | 0.594 |
| EPTB | 2.0 (1.7-2.3) | <0.000 | 1.3 (1.1-1.6) | 0.003 |
| Diagnosed using Xpert MTB/RIF |  |  |  |  |
| Yes | 1 |  | 1 |  |
| No | 1.7 (1.5-1.9) | <0.001 | 1.4 (1.2-1.6) | <0.001 |
| HIV status |  |  |  |  |
| Negative | 1 |  | 1 |  |
| Positive | 1.5 (1.3-1.6) | <0.001 | 1.5 (1.4-1.7) | <0.001 |

CI, confidence interval; DOT, directly observed therapy; TB, tuberculosis; EPTB, extra-pulmonary tuberculosis; HIV, human immunodeficiency virus; PTB, pulmonary tuberculosis; MTB RIF, mycobacterium tuberculosis and resistance to rifampin; uHR, crude hazard ratio; aHR: adjusted hazard ratio
