# Supplementary material for: Mortality among persons with tuberculosis in Zambian hospitals: A retrospective cohort study
Source: PLOS Glob Public Health. 2024 Jun 17;4(6):e0003329. doi: 10.1371/journal.pgph.0003329 (PMC11182540; doi:10.1371/journal.pgph.0003329)
Supplement: S3 Table — (DOCX) [file pgph.0003329.s005.docx]

**S3 Table. Probable causes of death among persons with TB Zambian hospitals: A sub-analysis of people living with HIV (2019)**

| **Probable cause of death on certificates**  **(n = 147)** | **#** | **%** | **Assigned probable causes of death**  **(n = 280)** | **#** | **%** |
| --- | --- | --- | --- | --- | --- |
| TB IRIS/Disseminated TB | 37 | 25.2 | TB IRIS/Disseminated TB | 53 | 18.9 |
| PTB | 30 | 20.4 | Sepsis | 49 | 17.5 |
| Sepsis/septic shock | 16 | 10.9 | End organ damage | 41 | 14.6 |
| TB meningitis | 15 | 10.2 | Anaemia | 34 | 12.1 |
| Anaemia | 12 | 8.2 | PTB | 26 | 9.3 |
| End organ damage | 11 | 7.5 | TB meningitis | 25 | 8.9 |
| Superadded pneumonia | 8 | 5.4 | Superadded pneumonia | 19 | 6.8 |
| Sequelae of TB | 6 | 4.1 | Other causes (malignancy, malaria, trauma) | 17 | 6.1 |
| Other CNS infections | 4 | 2.7 | Other CNS infections | 11 | 3.9 |
| Other (causes, malignancy, malaria, trauma) | 4 | 2.7 | Adrenal insufficiency | 2 | 0.7 |
| Aspiration pneumonia | 2 | 1.4 | Aspiration pneumonia | 2 | 0.7 |
| Adrenal insufficiency | 2 | 1.4 | Sequelae of TB | 1 | 0.4 |
| **Total** | **147** | **100** | **Total** | **280** | **100** |
