# Supplementary material for: Mortality among persons with tuberculosis in Zambian hospitals: A retrospective cohort study
Source: PLOS Glob Public Health. 2024 Jun 17;4(6):e0003329. doi: 10.1371/journal.pgph.0003329 (PMC11182540; doi:10.1371/journal.pgph.0003329)
Supplement: S4 Table — (DOCX) [file pgph.0003329.s006.docx]

**S4 Table. Probable causes of death among persons with TB Zambian hospitals: A sub-analysis of people not living with HIV (2019)**

| **Probable cause of death on certificates**  **(n = 60)** | **#** | **%** | **Assigned probable causes of death**  **(n = 219)** | **#** | **%** |
| --- | --- | --- | --- | --- | --- |
| PTB | 12 | 20.0 | Sepsis | 45 | 20.5 |
| End organ damage | 9 | 15.0 | TB IRIS/Disseminated TB | 36 | 16.4 |
| Superadded pneumonia | 8 | 13.3 | PTB | 31 | 14.2 |
| TB IRIS/Disseminated TB | 7 | 11.7 | End organ damage | 26 | 11.9 |
| Sepsis/septic shock | 7 | 11.7 | Other causes (malignancy, malaria, trauma) | 22 | 10.0 |
| TB meningitis | 5 | 8.3 | Adrenal insufficiency | 18 | 8.2 |
| Anaemia | 3 | 5.0 | Anaemia | 14 | 6.4 |
| Sequelae of TB | 3 | 5.0 | TB meningitis | 14 | 6.4 |
| Other CNS infections | 3 | 5.0 | Superadded pneumonia | 7 | 3.2 |
| Other (causes, malignancy, malaria, trauma) | 2 | 3.3 | Other CNS infections | 5 | 2.3 |
| Aspiration pneumonia | 1 | 1.7 | Aspiration pneumonia | 1 | 0.5 |
| **Total** | **60** | **100** | **Total** | **219** | **100** |
