# Supplementary material for: Mortality among persons with tuberculosis in Zambian hospitals: A retrospective cohort study
Source: PLOS Glob Public Health. 2024 Jun 17;4(6):e0003329. doi: 10.1371/journal.pgph.0003329 (PMC11182540; doi:10.1371/journal.pgph.0003329)
Supplement: S1 Fig — (DOCX) [file pgph.0003329.s007.docx]

**S1 Fig: Kaplan-Meier survival estimate for TB patients on treatment in Zambia in 2019 by sex, age, facility type, HIV status, method of diagnosis and type of TB**


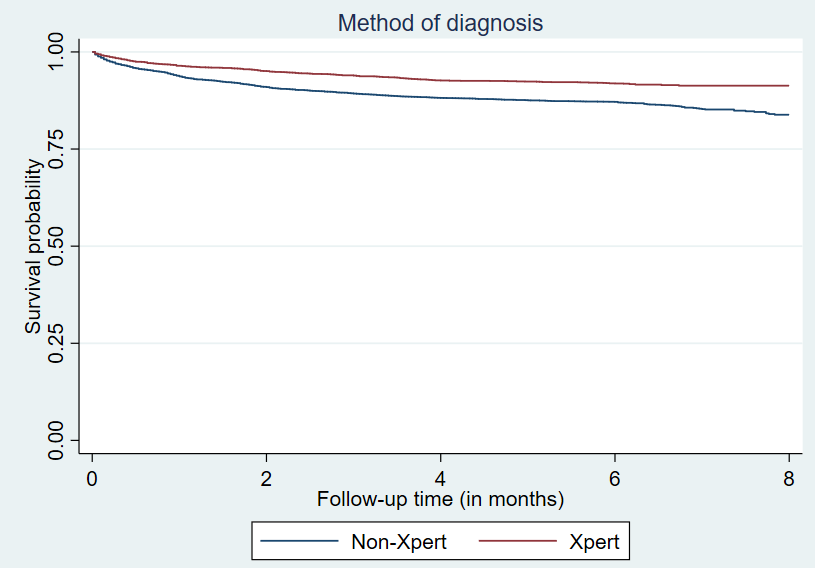

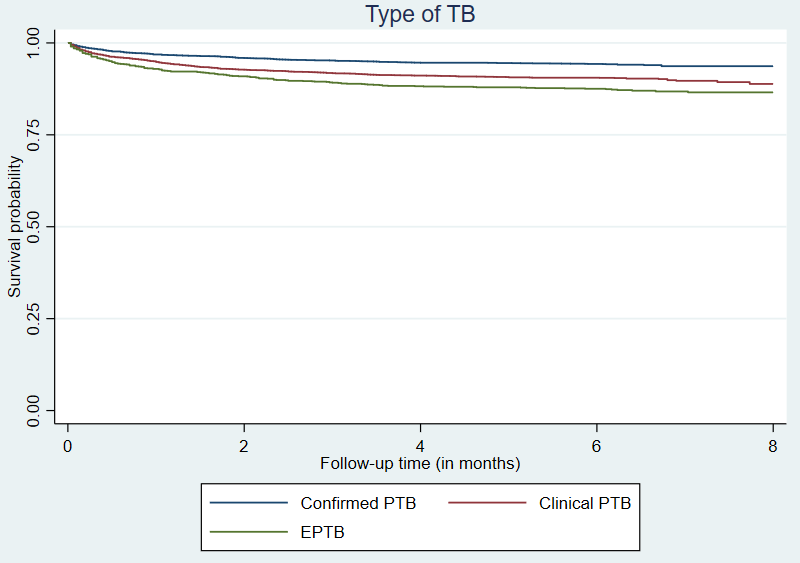

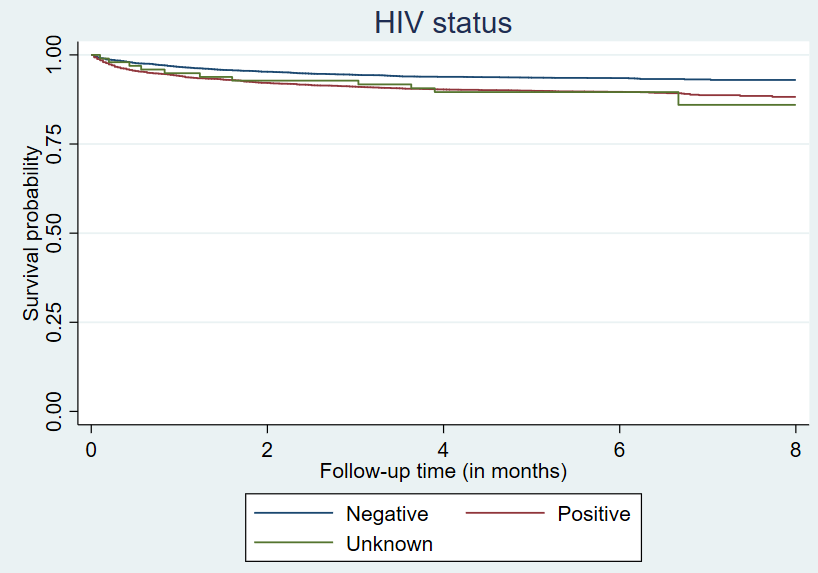

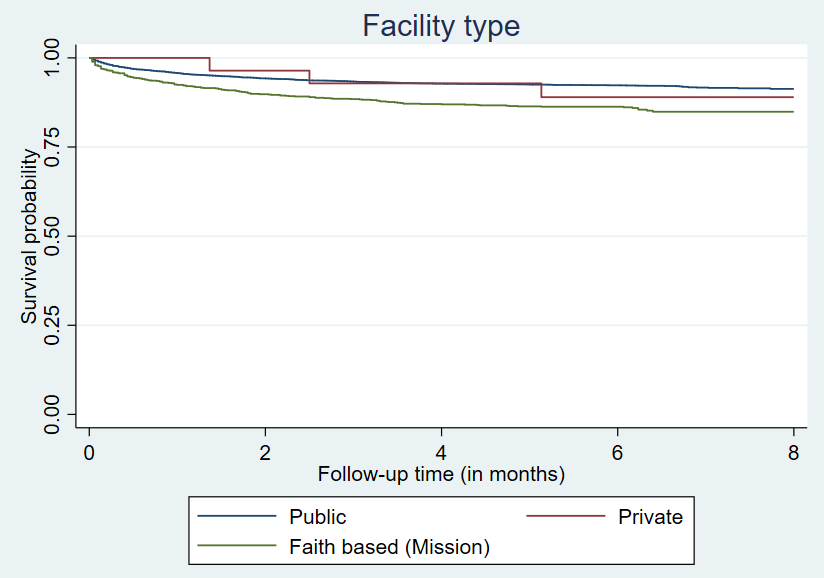

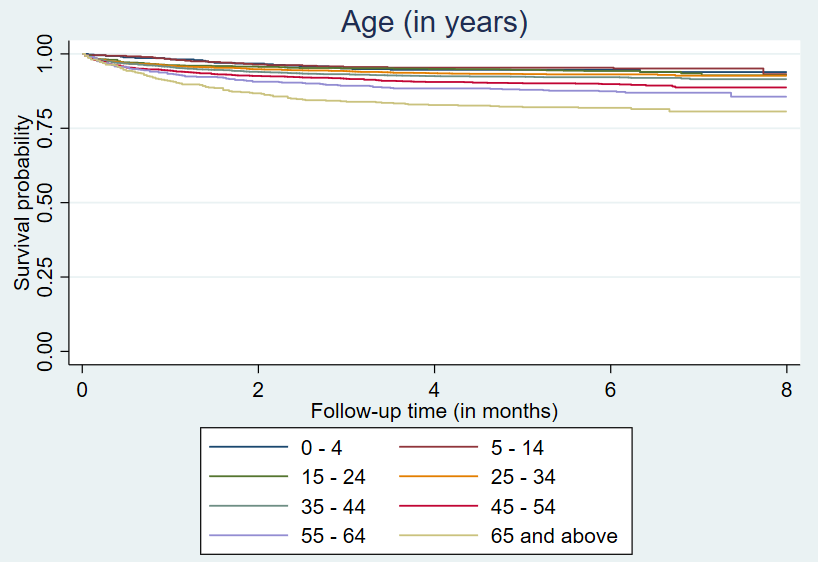

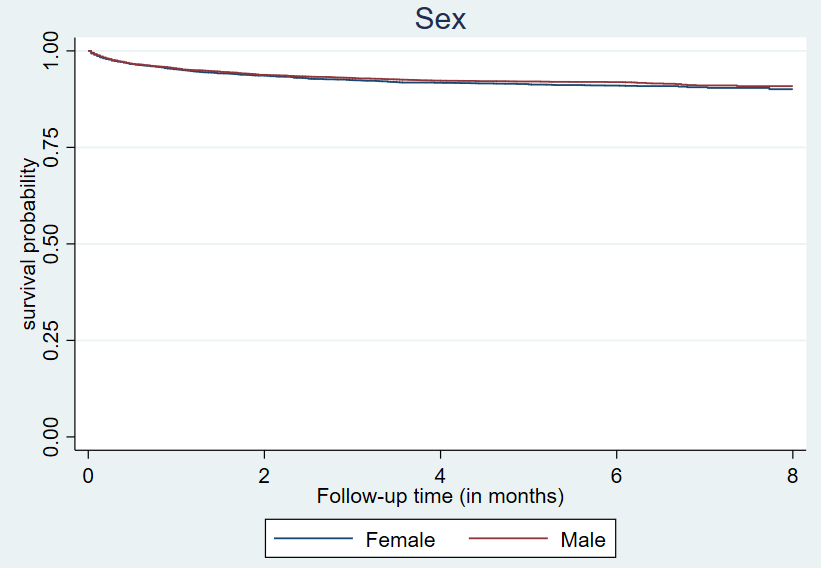


p-value = 0.001

p-value = 0.001

p-value = 0.001

p-value = 0.001

p-value = 0.684

p-value = 0.001
