# Supplementary material for: Mortality among persons with tuberculosis in Zambian hospitals: A retrospective cohort study
Source: PLOS Glob Public Health. 2024 Jun 17;4(6):e0003329. doi: 10.1371/journal.pgph.0003329 (PMC11182540; doi:10.1371/journal.pgph.0003329)
Supplement: S2 Fig — (DOCX) [file pgph.0003329.s008.docx]

**S2 Fig: Comorbid condition or end organ damage as a cause of death among persons with TB in Zambia (2019), n = 499**
